# Seminal Analysis and Testicular RNA Sequencing of a Ram with Low Fertility

**DOI:** 10.3390/ani16152352

**Published:** 2026-08-01

**Authors:** María Teresa Tejedor, Rosaura Pérez-Pe, Luis Vicente Monteagudo, Melissa Carvajal-Serna, Victoria Peña-Delgado, Angel Macías, Elena Martín, Adolfo Laviña

**Affiliations:** 1 Departmento de Anatomia, Embryologia y Genetica Animal, Faculty of Veterinary Sciences, Universidad de Zaragoza, 50013 Zaragoza, Spain; ttejedor@unizar.es; 2 Centro de Investigacion Biomedica en Red Enfermedades Cardiovaculares (CIBER CV), Faculty of Veterinary Sciences, Universidad de Zaragoza, 50013 Zaragoza, Spain; 3 Grupo BIOFITER-IUCA, Departamento de Bioquímica y Biología Molecular y Celular, Universidad de Zaragoza, 50800 Zaragoza, Spain; rosperez@unizar.es (R.P.-P.); melissac@unizar.es (M.C.-S.); vpdelgado@unizar.es (V.P.-D.); 4 Asociación Nacional de Criadores de Ganado Ovino de la Raza Rasa Aragonesa (ANGRA), 50820 Zaragoza, Spain; angel@rasaaragonesa.com (A.M.); elena@rasaaragonesa.com (E.M.); adolfo@rasaaragonesa.com (A.L.)

**Keywords:** ovine, fertility, semen quality, transcriptome, differently expressed genes

## Abstract

One ram of the Rasa Aragonesa breed (M) exhibited low reproductive performance throughout his reproductive life (ewes that lambed/ewes inseminated: median: 0.57, interquartile range: 0.35; lambs born/ewes inseminated: 0.96 ± 0.513). In this exploratory case comparison, our objective was to determine the causes of M’s reproductive problems. Another ram (O) was chosen as the reference animal due to its history of high fertility and fecundity (fertility: median: 0.73, interquartile range: 0.33; fecundity: 1.22 ± 0.523). Semen samples from both males were submitted to a complete quality study. M exhibited lower sperm concentration, reduced membrane integrity, altered ROS/live–dead sperm profiles, increased phosphatidylserine inversion, and reduced DNA integrity. RNA sequencing, mapping against the reference genome (*Ovis aries*, GCF_016772045.2), differential gene expression, and GO enrichment analysis were performed on testicular biopsies of M and O. Despite the limitations of this study, which is based solely on two rams with extreme reproductive performances, it was detected that the *KRT2* gene was downregulated in M with respect to O, with possible deleterious effects on membranes. Since only two single individuals were considered, this transcriptomic finding should be interpreted as hypothesis-generating rather than confirmatory. Therefore, for *KRT2* to be considered a likely candidate to explain the observed differences in sperm quality and fertility outcomes, a larger number of animals with either high or low fertility should be considered, and validation using qRT-PCR should be performed.

## Simple Summary

Low fertility is a significant problem in animal production that can result in significant economic losses. Male fertility is particularly important in artificial insemination since each male must fertilize a large number of females. One ram of the Rasa Aragonesa sheep breed (M) showed lower fertility (ewes that lambed/ewes inseminated: median: 0.57, interquartile range: 0.35) and fecundity (lambs born/ewes inseminated: 0.96 ± 0.513) values than another (O) that was considered to represent a normal male (fertility: median: 0.73, interquartile range: 0.33; fecundity: 1.22 ± 0.523). In this exploratory case comparison, semen samples from both males were submitted to a complete study of semen quality; semen from M was shown to be of lower quality than that from O. Using testicular biopsies from M and O, RNA sequencing, differential gene expression analysis, and GO enrichment analysis were performed in order to identify differentially expressed genes that could explain these differences. The *KRT2* gene was downregulated in M compared to O, with potential effects on membranes. Since only two single individuals were considered, this transcriptomic finding should be interpreted as hypothesis-generating rather than confirmatory. Therefore, for *KRT2* to be considered a likely candidate to explain the observed differences in sperm quality and fertility outcomes, a larger number of animals with either high or low fertility should be considered, and validation using qRT-PCR should be performed.

## 1. Introduction

The range of results from sheep genetic improvement programs is presently acquired mainly via artificial insemination (AI) procedures rather than through natural mating, in spite of the difficulties of this procedure in the ovine species that stem mainly from the structure of the ewe cervix and the poor results obtained when frozen–thawed semen is used [1]. In order to obtain good AI results, it is critical to use spermatozoa with high fertilizing potential, but this implies a need to know the fertilizing ability of a seminal dose in advance [2]. The standard analysis of semen, based exclusively on progressive motility, normal morphology, intact plasma, and acrosomal membranes, does not allow for accurate prediction of fertility; more advanced evaluation procedures, such as the non-adherence of the sperm or zona-free hamster oocyte penetration, are currently proposed in human fertility. Moreover, the selection of different fractions of ejaculate is known to produce better results in assisted reproductive technologies [2]. Recently, Mendoza et al. [3] found that the percentage of non-capacitated spermatozoa with intact membranes and intact DNA was significantly correlated with fertility and fecundity following artificial insemination in sheep. Furthermore, omics approaches could be useful diagnostic tools for assessing male infertility, and it has been shown that the differential expression of sperm RNAs is related to fertility in humans and other species; differences in the micro-RNA profiles in boars and bulls have been shown to exist when low- and high-fertility males are compared [4,5]. Regarding ovine species, a transcriptomic analysis performed in three different breeds (Merino, Dohne, and Poll Dorset) concluded that 754 genes are differently expressed in the different breeds and also when ejaculates of different qualities (as evaluated by means of computer-assisted semen analysis) are contrasted; these genes could therefore play significant roles in physiological functions related to ovine reproduction, including fertilization. As a consequence, analysis of the spermatozoa transcriptome has potential as a tool in the improvement of ram reproductive efficiency or, at least, in the selection of the reproducers providing the most competent spermatozoa [6].

Thousands of RNAs, even mRNAs, are present in spermatozoa, as determined by array hybridization, RT-PCR, and in situ hybridization [7,8]. These mRNAs are synthesized during spermatogenesis and stored in mature spermatozoa, so that not only the paternal genome but also a unique suite of paternal mRNAs is provided to the zygote [9]. Protein synthesis is thought to be performed by mitochondrial [10] and cytoplasmic ribosomes [11]. The possible role of cytoplasmic ribosomes is a matter of controversy; in a recent study, although the protein synthesis activity of cytoplasmic ribosomes could not be excluded, this activity was demonstrated in mitochondrial ribosomes. The inhibition of this activity by chloramphenicol results in reduced production of at least two proteins and, as a consequence, in impaired sperm motility [12]. However, the mechanism by which nuclear-encoded proteins are synthesized by mitochondrial ribosomes is still unknown.

ANGRA (Asociación Nacional de Criadores de Ganado Ovino Selecto de Raza Rasa Aragonesa) is the breeders’ association responsible for the management and genetic improvement of the Rasa Aragonesa sheep breed in Spain. This breed is reared for meat production and farmed under semi-extensive conditions; the main product is a light lamb protected under the commercial designation “Ternasco de Aragón”. Carcass quality and reproduction indexes are among the characteristics that are of greatest significance to improving farms profits, and both have been targeted by genetic improvement programs over the last forty years. As is the case in other animal species and breeds, AI is currently preferred to animal mating as the main way of spreading genetic improvement to a high proportion of herds. ANGRA has in fact extended AI to over 172 farms since 1989. Continuous oversight of the factors affecting AI efficiency is a critical aspect of this procedure. ANGRA maintains in-depth and direct statistical supervision of the results of inseminations in an effort to identify the factors that could improve the protocols applied in the field at each farm. For instance, Tejedor et al. [13] published the conclusions drawn from the analysis of the results of AI in 58.602 ewes (5.945 AI lots at 172 farms). As a result, a program titled FERTIA modified several aspects of the AI protocol in order to increase fertility. Even an anti-retrograde flow device (DARIO) for sheep cervical artificial insemination was designed and used, resulting in a significant increase in both fertility and fecundity rates, along with a marginal increase of 9.05 pregnancies per 100 inseminated ewes and of 0.15 lambs per insemination [14].

Thanks to the continuing oversight of the artificial insemination success rate in Rasa Aragonesa, the technical services of ANGRA detected that one of the available rams exhibited a relatively low reproductive performance throughout its reproductive life when compared with the mean values reported by the organization for the whole population of AI rams. It is important to note that routine inspections had not previously detected any defects in the semen doses obtained from this male.

Our objective was to determine the causes of this poor performance; therefore, we carried out both a complete study of semen quality and RNA sequencing and compared the results to those of another ram with high fertility and fecundity values under the same production conditions. This study is therefore an exploratory case comparison in Rasa Aragonesa rams. Being aware of our study’s limitations, stemming from the small number of animals analyzed (only one low-fertility ram was available), differentially expressed genes (DEGs) preliminarily detected in this study could be potential markers for infertility in rams. However, since only one individual per group was considered based on fertility (low fertility vs. high fertility), the transcriptomic findings should be interpreted as hypothesis-generating rather than confirmatory. A study with a larger number of animals presenting a low fertility index, contrasted against a set of normally fertile rams, would be necessary to corroborate the results obtained in the present study.

## 2. Materials and Methods

The authors did not use any generative AI (artificial intelligence) or AI-assisted technologies in the process of finalizing this manuscript.

Unless otherwise stated, all reagents were purchased from Sigma-Aldrich (St. Louis, MO, USA).

### 2.1. Animals

This study complied with the ARRIVE guidelines [15]. All animal procedures were performed in accordance with the Spanish Animal Protection Regulation RD1201/05 [16], which conforms to European Union Regulations (Directive 2010/63) [17]. The Ethics Committee of the University of Zaragoza approved the testicular biopsy protocol (PI25/25, 25 March 2025). Approval from this Ethics Committee was not a prerequisite for the semen analysis because semen was obtained by technicians at the artificial insemination station, and an ANGRA veterinarian carried out the artificial insemination (AI) according to their breeding schedule and provided data about AI characteristics and results.

The Rasa Aragonesa breed is characterized by a subconvex profile, medio-linear proportions, and variable size according to the areas it inhabits. Adult rams weigh 50–85 kg and ewes 35–50 kg. It occupies the greater part of the Ebro basin (Northeast Spain), and this breed stands out for its ruggedness, gregarious instinct, pasturing ability, and adaptability to the harsh environment in which it is raised. It is reared mainly for its meat; it produces the commonest slaughter lamb in Aragon (characterized by tender meat with initial intermuscular fat infiltration, highly juicy and soft in texture, and with a very pleasant bouquet; carcass weight: 8.5–11.5 kg). Additionally, the Rasa Aragonesa produces a fleece of medium-quality wool, white in color, and with square strands (diameter (microns): 25–28; length (cm): 6–7; fleece weight (kg): 1.8–3; yield upon deep washing (%): 43–47) [18]. Currently, the population consists of 285,816 individuals distributed across 398 livestock farms [19].

Two mature rams of the Rasa Aragonesa breed were investigated. One of them (designated M) was identified by the ANGRA technical services as it registered relatively low fertility and fecundity throughout its reproductive life. It was compared against O, a ram with high fertility results, selected as a reference animal to highlight possible differences in semen quality and RNA sequencing characteristics. Both males were compared at the same reproductive age; their use as artificial insemination males began at around two years of age. During the insemination period considered in this study, the weights of both males ranged between 70 and 80 kg, with an average body condition score of 3 (2.5–3.5). Body condition was measured using a scale of 1 to 5 (where 1 is very thin/emaciated and 5 is very fat/obese) [20].

Both males were housed under the same conditions at CENSYRA (Regional Centre for Animal Selection and Reproduction) in Zaragoza, Spain (41°39′06.00″ N 0°52′58.98″ W). Both males were also fed the same diet: a dry mixture (Rum Paca Machos) of cereal straw, cereal (usually barley), concentrate (soybean cake or gluten-based feed), corrector, and molasses (as a binder for the mixture).

Semen from M was used for AI in 90 batches from 37 farms (between 9 January 2015 and 26 August 2022; 8.06 ± 4.851 ewes/batch), while O’s semen was used for AI in 65 batches from 35 farms (between 10 June 2020 and 20 October 2023; 7.68 ± 3.251 ewes/batch).

These batches were not specifically chosen for the comparison of these males, but rather constitute the complete history of their reproductive life and correspond to the normal functioning of the farms to which they belong. The ewes in these batches were between 2 and 6 years old and, for the most part, had body condition scores between 2.49 and 3.49.

All AI operations were carried out by the ANGRA technical team, and all ewes were owned by member farms of ANGRA. The data provided concerned fertility (the proportion of inseminated ewes that lambed), fecundity (the number of lambs born per ewe inseminated), and prolificacy (the number of lambs born per ewe that lambed).

### 2.2. Semen Collection and Quality Analyses

The semen samples were collected by the station technician using an artificial vagina in adherence with the regular collecting schedule at the AI station. Semen analyses were performed on 7 samples from M (collected between 15 June 2020 and 20 September 2022) and 13 samples from O (collected between 21 September 2020 and 29 August 2023).

A routine semen assessment was conducted immediately after collection, measuring the ejaculate volume, concentration (using a spectrophotometer), mass motility (scored 0–5 via optical microscopy), and the proportion of motile spermatozoa (evaluated with a CASA system, AI Station, Sperm Analysis Technologies S.L, Buñol, Valencia, Spain). Following this initial check, semen was diluted to 1.6 × 10^9^ spermatozoa/mL with INRA 96 (IMV Technologies, L’Aigle, France). The diluted samples were then loaded into 0.5 mL straws and refrigerated at 15 °C for transport to the farms where AI was performed or to the laboratory of the University of Zaragoza where seminal doses were extensively analyzed. Semen quality analyses included sperm motility, morphology, membrane integrity, intracellular levels of reactive oxygen species (ROS), phosphatidylserine (PS) inversion, DNA fragmentation, and capacitation status.

Motility parameters were measured using a computer-assisted CASA system (ISAS 1.0.4; Proiser SL, Valencia, Spain) as previously described [21]. For each sample, five videos were recorded at 25 frames/s for 1 s. Spermatozoa with straightness (STR) > 80% were considered progressively motile, and those with curvilinear velocity (VCL) > 75 μm/s were considered rapid spermatozoa.

Sperm morphology was assessed by bright-field microscopy after staining with eosin and nigrosine, as described in Peña et al. [22]. At least 200 spermatozoa were analyzed per sample at 1000× magnification to determine the percentage of normal morphology cells. Sperm cells that showed a detached head, bent tail, coiled tail, or proximal or distal droplet were considered abnormal [23].

Cell membrane integrity (viability) was evaluated using carboxyfluorescein diacetate (CFDA) and propidium iodide (PI) adhering to a modification of the procedure described by Harrison and Vickers [24]. ROS were measured using H_2_DCFDA combined with PI to differentiate live and dead spermatozoa with high or low ROS levels [25]. FITC-Annexin V (Thermo Fisher Scientific, Waltham, MA, USA) was used to detect PS translocation and combined with PI to differentiate between membrane-intact and damaged cells, with or without PS translocation. To detect potential DNA strand breaks related to apoptosis, the TUNEL (terminal transferase mediated dUDP nick-end labeling) assay was applied by using the In Situ Cell Death Detection Kit, fluorescein (Roche, Mannheim, Germany) [26]. DNA was considered to be intact in TUNEL-negative spermatozoa. All these techniques were carried out as described in Peña et al. [22], and samples were evaluated by flow cytometry as explained therein.

The chlortetracycline (CTC) assay was used to evaluate capacitation status [27,28]. Stained spermatozoa were examined under a Nikon Eclipse E-400 microscope with epifluorescence illumination using a V-2A filter (×1000) (Nikon, Tokyo, Japan), and at least 200 cells per slide were classified as follows [29]: non-capacitated (NC, even distribution of fluorescence on the head, with or without a bright equatorial band), capacitated (C, with fluorescence in the anterior portion of the head), and acrosome-reacted cells (showing no fluorescence on the head).

### 2.3. Statistical Comparisons

IBM SPSS Statistics 26.0 software (IBM Corp., Armonk, NY, USA) was used to compare AI characteristics, results, and semen quality of M and O. The Pearson chi-squared test was used for comparisons of categorical variables. The normality of the distribution was tested by Shapiro–Wilk test for quantitative variables. Descriptive statistics used mean and SD (standard deviation) for normally distributed and median and IQR (interquartile range, Q3–Q1) for non-normally distributed variables. Student’s *t*-test, or alternatively Mann–Whitney’s U test, was applied to compare quantitative variables. The significance level was set at *p* ≤ 0.05.

### 2.4. Testicular Biopsy

The testicular biopsies were carried out in April 2025 by ANGRA veterinarians using a modification of the TESA (testicular sperm aspiration) technique widely used in humans [30]. Before the biopsy, the incision zone was locally anesthetized by subcutaneous injection of 12 mL lidocaine (2%) (Lidor 2% 50 mL, VetViva Richter GmbH, Durisolstrasse 14, Wels, Austria). Once the testicle was exposed, individual samples were obtained using single-use biopsy needles (Tru cut^®^, Angiotech Pharmaceuticals TM, Vancouver, BC, Canada). These needles measure 11.4 cm in length, although only the first 4 cm were used in the testicular puncture to allow for the collection of samples approximately cylindrical in shape, with a maximum length of 20 mm, a diameter of 1.5 mm, a maximum volume of 38 mm^3^, and a maximum weight of 38–40 mg.

### 2.5. RNA Sequencing

The PureLink^®^ RNA Mini Kit (Thermo Fisher Scientific, Waltham, MA, USA) was used for RNA extraction from testicular samples. RNA sequencing and statistical analyses were carried out by STAB VIDA (Lda, Caparica, Portugal). Next-generation sequencing (NGS) was applied to RNA sequencing by using the Illumina^®^ HiSeq^®^ system (Illumina, Inc., San Diego, CA, USA). Analysis of the generated raw sequence data was carried out using CLC Genomics Workbench 12.0.3 and OmicsBox v 3.1.11 (Qiagen N.V., Venlo, The Netherlands). The bioinformatics analysis started with the trimming of raw sequences to generate high-quality data only. The trimming parameters applied had the following limit thresholds: ambiguous limit, 2 nucleotides; quality limit, 0.01 (error probability); minimum number of nucleotides in reads, 30 nucleotides; short reads were discarded. The high-quality sequencing reads were mapped against the reference genome *Ovis aries* (GCF_016772045.2), using the parameters length fraction = 0.8 and similarity fraction = 0.8 (at least 80% of the alignment must match the reference sequence and identity should be at least 80% for the read to be included in the mapping analysis). The mapping results served to determine the gene expression levels based on TPM (transcripts per million), which is a variant of the RPKM (reads per kilobase of exon model per million mapped reads) method, calculated as

TPM = RPKM × 10^6^/∑RPKM

where RPKM = total exon reads/mapped reads [millions] × exon length [Kb].

Differential Expression for RNA-Seq is a multi-factorial statistical analysis tool based on a negative binomial model. It uses a generalized linear model approach influenced by the multi-factorial EdgeR method. The differentially expressed genes (DEGs) showed significant changes when M was compared with O. Fold change and FDR (false discovery rate) were calculated for each considered gene log_2_. The conditions were log_2_ fold change ≥ 2 or ≤−2, and FDR (false discovery rate) *p*-value ≤ 0.05. When the FDR *p*-value was ≤0.05, if the log_2_ fold change was ≤−2, a DEG was considered to be downregulated, while a DEG was upregulated if the log_2_ fold change was ≥2 [31]. Results are shown in a volcano plot.

Several databases were screened for functional annotation of DEGs (GeneCard; AmiGO2; MGI: Mouse Genome Informatics, and IMPC: International Mouse Phenotyping Consortium) [32,33,34,35]. GO annotation and enrichment analysis was also carried out.

## 3. Results

Data on the characteristics and results of AI, including the age and body condition of the ewes in the batches, were supplied by ANGRA and are shown in Table 1, with AI batch as the study unit. There were no significant differences between the rams in terms of the conditions under which artificial insemination was performed or the age and body condition of the females.

Table 2 shows the semen characteristics of both rams. M exhibited significantly lower values for sperm concentration, percentage of spermatozoa with membrane integrity (%), live spermatozoa with low ROS values, and spermatozoa with intact DNA (%). M also exhibited significantly higher values for dead spermatozoa with PS inversion (%). No significant differences in ejaculate volume, motility, morphological abnormalities, or capacitation status were found between the two rams. Fertility and fecundity values were lower for M, while no difference was found for prolificacy.

Table 3 shows the metrics of RNA-sequencing from O and M rams. Both the volume of reads and the depth of coverage suggested adequate statistical robustness for both transcript reconstruction and gene quantification.

**Figure 1:**
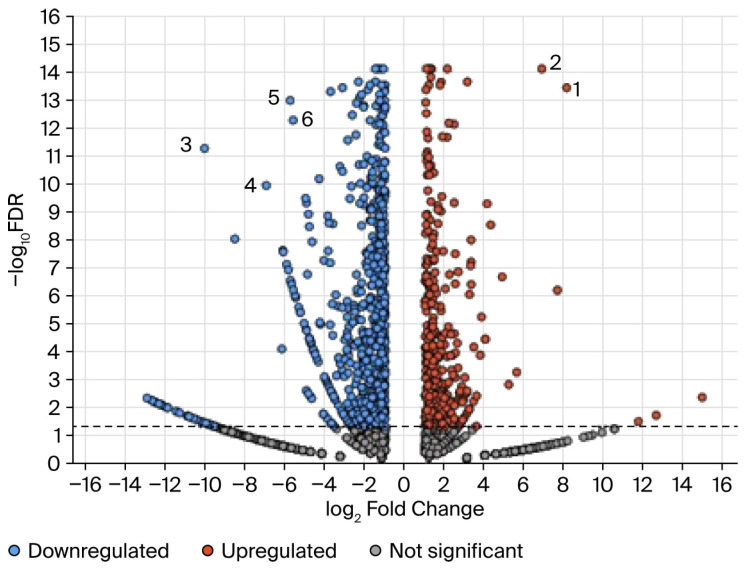
Volcano plot of studied genes, represented by dots. Orange and blue dots indicate upregulated and downregulated DEGs, respectively, and gray dots indicate non-differentially expressed genes. The dotted lines represent the cut-off values of the differential expression (−log_10_FRD = 1.30). 1: *RPS12*; 2: *LOC114111581 (FTH1)*; 3: *KRT2*; 4: *KLK7*; 5: *C2CD4C*; 6: *SLC6A14*.

In the RNA sequencing, 35,117 genes met the specified criteria, with 1306 DEGs (865 downregulated and 441 upregulated genes in M compared to O). Figure 1 shows the volcano plot of the studied genes. As can be seen, a high value of log_2_ fold change was not always accompanied by a high value of −log_10_FDR.

Table 4 shows the downregulated DEGs with both most extreme (negative) log_2_ fold change and −log_10_FDR values. Four genes meet these conditions: *KRT2* (*Keratin 2*; chromosome 3), *KLK7* (*Kallikrein-related peptidase 7*, chromosome 14), *C2CD4C* (*C2 Calcium Dependent Domain Containing 4C*, chromosome 5), and *SLC6A14* (*Solute Carrier Family 6 Member 14*, chromosome X).

Table 5 shows the upregulated DEGs with both most extreme (positive) log_2_ fold change and −log_10_FDR values. Only two genes meet these conditions: *RPS12* (*Ribosomal protein S12*, chromosome 8) and *LOC114111581* (*FTH1: Ferritin heavy chain-like*, chromosome X). Additionally, data about length of read (bp), reads, TPM RPKM, and relative RPKM rate are shown in both tables.

GO enrichment analysis only showed significant results for *KRT2* (see Table 6). The results suggest a relationship between this gene, the cytoskeleton, and the filaments that form it.

## 4. Discussion

The significant differences in sperm quality measurements indicate that the sperm quality of ram M was lower. Important confounding factors in the comparison of both males were controlled where possible. Although the historical records correspond to different (but overlapping) periods for the two males, as do the dates of semen analysis, both males were of a similar age during the periods considered. As described in Section 2, the body weight, body condition, and diets of the two rams were similar. These differences would explain the observed differences in the reproductive performance of the two rams. However, prolificacy depends on the ewe’s ovulation, and therefore the lack of differences between the rams was not an unexpected result [36].

Sperm quality and fertility are related; the number, motility, morphology, and viability of sperm cells are critical for successful fertilization of an egg and subsequent pregnancy but predicting fertility through the in vitro evaluation of sperm quality remains a challenge [37]. Sperm concentration influences the likelihood of pregnancy in sheep [38]. Membrane integrity is crucial for successful fertilization [39,40,41].

Previous studies have found the inclusion of non-conventional sperm quality parameters, such as DNA integrity and capacitation status, to be important in the prediction of fertility in ovine species [3]. In accordance with this, the low-fertility ram (M) in this study demonstrated significantly lower percentages of spermatozoa with intact DNA (%) than the ram with good reproductive performance (O). In contrast, no differences in the capacitation status were observed between M and O. However, differences were detected in the percentage of live sperm with low ROS values, indicating greater oxidative stress in spermatozoa from M, which is consistent with the study by Del Olmo et al. [42], which concludes that detecting ROS production could be a useful procedure for predicting the fertility of ram spermatozoa. Moreover, M showed significantly higher values for dead spermatozoa with PS inversion. Several studies have demonstrated that PS exposure may be used as an indicator of sperm quality and that the removal of EPS spermatozoa may enhance fertility potential in assisted reproductive techniques [43].

In terms of data volume, RNA sequencing from both rams exceeds the standards recommended by international consortia for most RNA sequencing analyses (recommended standard for reads: 20–30 million; covering 30–50) [44,45].

To delve deeper into the differences between M and O, RNA sequencing was carried out. The most complex transcriptome is expressed by male germinal cells [46]; functional sperm needs multiple RNA pathways to protect the integrity of both spermatogenesis and the genome from stressors. For this reason, several of the DEGs detected so far have no clearly defined functions related to spermatozoa. With only one animal per group, one of the limitations of this study, biological dispersal could not be reliably estimated, and classifying genes solely by extreme log2 fold change proved unstable. In fact, the volcano plot showed DEGs with extreme values of log2 fold change but showed low values of −log_10_FDR. Despite this, the shape of the volcano plot and the GO enrichment analysis would suggest that downregulated *KRT2* may play a role in the low fertility values of M.

In humans, according to GeneCards, the *KRT2* gene is primarily expressed in the skin, but it is also expressed in the testicle (TPM: 0.31). As indicated in Section 2, the biopsy technique (ESA) consists of direct aspiration from the testicle once exposed; therefore, contamination from the skin can be ruled out. Cytoskeletal elements are fundamental to spermatogenesis processes and, therefore, crucial for male fertility [47]. Four elements compose the eukaryotic cytoskeleton: actin filaments (microfilaments [48], microtubules [49], intermediate filaments [50], and septin proteins [51]). Several keratins and intermediate filaments participate in important spermatogenesis processes. Acrosome biogenesis is facilitated by the acroplaxome, a dense F–actin/keratin–5 cytoskeletal structure that anchors the acrosome to the nucleus via interaction between this keratin and the intermediate filament network in the nuclear envelope. Acroplaxome also modulates sperm head shape [52]. The sperm flagellum is formed by the axoneme, sperm-specific accessory structures (including keratin-like outer dense fibers (ODFs), a mitochondrial sheath, and a fibrous sheath [53]. The compaction and elongation of the spermatid nucleus is marked by the formation of the manchette around the distal half of the nucleus, stabilized by a dense tubulin/keratin 9-based perinuclear ring [54,55].

In addition, cryopreservation has been shown to alter the proteome of ram sperm, possibly leading to reduced sperm fertility after thawing. Several keratins (*KRT5*, *KRT1*, *KRT14*, and *KRT7*) were included in the protein set, with a significantly lower expression in the fresh samples compared to the frozen–thawed samples [56]. These findings contribute to highlighting the important role keratins play in male fertility.

There is a connection between the membranes and the microtubule cytoskeleton [57]; therefore, the downregulated activity of the *KRT2* gene could be a possible or potential explanation for the lower percentage of membrane integrity in ram M. Functional validation would be necessary to confirm this potential relationship.

Beyond the established role of keratins in spermatogenesis, specific keratins are instrumental in genital organ development. There are no specific studies on this topic in sheep, but there are in laboratory species that serve as models for mammals. In mice, a mutation of *KRT2* (*Krt2^tm1b^^(KOMP)Wtsi^*) is related to both abnormal testis morphology and small testis [34]. In rats, KRT33B facilitates penis growth by providing an essential cytoskeletal scaffold that ensures structural integrity and mechanical resilience during rapid growth phases [58]. Furthermore, the gene encoding Keratin 33B has been linked to abnormal testis morphology and reduced testis size in mice, according to MGI and IMPC records. Similarly, *KRT27* and *KRT35* have both been implicated in abnormal seminal vesicle morphology in mouse models [34,35]. However, these findings provide supporting biological context rather than direct evidence for the same mechanism in sheep.

Validation of the candidate gene using qRT-PCR could not be performed, which constitutes another limitation of this study. On the other hand, it is not possible to overlook one of the main limitations of this study; only two individuals were considered, which entails inherent statistical limitations in the estimating differential expression and false discovery rates. Therefore, this transcriptomic finding should be interpreted as hypothesis-generating rather than confirmatory. A study with a larger number of animals of either high or low fertility and subsequent validations would be necessary to corroborate the results of this study.

## 5. Conclusions

When compared with a ram with excellent fertility (O), a ram with low fertility (M) showed significantly lower sperm concentration, reduced membrane integrity, altered ROS/live–dead sperm profiles, increased PS inversion, and reduced DNA integrity. These sperm characteristics could explain the observed low fertility. On the other hand, RNA sequencing showed *KRT2* to be downregulated in M compared to O, which could have a potential effect on membranes. Since only two single individuals were considered, this transcriptomic finding should be interpreted as hypothesis-generating rather than confirmatory. Therefore, for *KRT2* to be considered a likely candidate to explain the observed differences in sperm quality and fertility outcomes, a larger number of animals with either high or low fertility should be considered, and validation by qRT-PCR should be performed.

## Figures and Tables

**Table 1:** Characteristics and results of artificial insemination for both rams (M and O), including the characteristics of the ewes in the batches.

Variable	Category	Ram		*p*-Value
		O	M	
AI technician	1	26/65 (40.0%)	32/89 (36.0%)	0.703
	2	9/65 (13.8%)	10/89 (11.2%)	
	3	30/65 (46.2%)	47/89 (52.8%)	
Age of ewes (years)	2.0–3.9	20/64 (31.3%)	35/88 (39.8%)	0.364
	4.0–6.0	44/64 (68.8%)	53/88 (60.2%)	
Body condition of ewes	0–2.49	6/64 (9.4%)	7/85 (8.2%)	0.062
	2.49–3.49	57/64 (89.1%)	68/85 (80.0%)	
	≥3.50	1/64 (1.6%)	10/85 (11.8%)	
Flushing		44/65(67.7%)	60/86 (69.8%)	0.924
Fertility		0.7273 [0.33]	0.5714 [0.35]	0.001
Fecundity		1.2195 ± 0.52308	0.9586 ± 0.51319	0.002
Prolificacy		1.8167 [0.42]	1.7143 [0.50]	0.202

Data are count/n (%), mean ± SD, or median [interquartile range, IQR].

**Table 2:** Semen characteristics for both rams.

Variable	Ram				*p*-Value
	O		M		
	n	Mean ± SD; Median [IQR]	n	Mean ± SD; Median [IQR]	
Volume (mL)	11	1.100 [0.3]	5	1.000 [0.6]	0.115
Total motility (%)	13	88.54 ± 7.601	7	86.43 ± 3.409	0.405
Progressive motility (%)	13	37.15 ± 8.019	7	43.43 ± 6.876	0.098
Rapid spermatozoa (%)	13	74.23 ± 14.048	7	75.14 ± 4.947	0.835
Sperm concentration (×10^6^)	11	4810.91 ± 888.611	5	3520.00 ± 908.213	0.030
Membrane integrity (% CFDA+/IP- spermatozoa)	13	87.946 ± 5.2263	7	65.429 ± 8.1158	<0.001
Live spermatozoa with low ROS values (PI-/H_2_DCFDA-) (%)	13	80.569 ± 5.0686	7	62.314 ± 7.8980	<0.001
Dead spermatozoa with PS inversion (%)	13	9.377 ± 7.1082	7	23.214 ± 13.2526	0.033
Non-capacitated sperm (NC, %)	13	89.15 ± 7.221	7	83.71 ± 9.032	0.199
Capacitated sperm (C, %)	13	7.77 ± 5.215	7	13.86 ± 9.209	0.144
Acrosome-reacted sperm (AR, %)	13	3.00 [3]	7	2.00 [1]	0.817
TUNEL-negative sperm (intact DNA) (%)	13	91.492 ± 6.3261	7	87.100 ± 2.8729	0.048
Normal morphology (%)	11	91.45 ± 5.574	6	95.17 ± 6.145	0.224
Sperm with loose head (%)	11	1.00 [4]	6	0.00 [1]	0.404
Sperm with proximal cytoplasmic drop (%)	11	1.00 [2]	6	0.00 [2]	0.301
Sperm with distal cytoplasmic drop (%)	11	0.00 [1]	6	0.00 [0]	0.149
Sperm with bent tail (%)	11	2.00 [5]	6	2.50 [5]	0.976
Sperm with coiled tail (%)	11	1.00 [2]	6	0.00 [1]	0.078

Data are count/n (%), mean ± SD, or median [interquartile range, IQR].

**Table 3:** RNA sequencing metrics for rams O and M.

Ram	Average Length of Reads (bp)	Reads	Obtained Data (Gb/Sample)	Covering	Average TPM
O	5161.14	48,556,723	252.49	93.52	13.04
M	5541.03	63,440,952	348.92	129.23	13.04

TPM: Transcripts per million.

**Table 4:** Downregulated DEGs with most extreme (negative) log_2_ fold change and −log_10_FDR values.

Gene ID	Gene Name		Ram O					Ram M					Log_2_ Fold Change	−Log_10_FDR
		Chr. Number	Length of Read (bp)	Reads	TPM	RPKM	Relative RPKM	Length of Read (bp)	Reads	TPM	RPKM	Relative RPKM		
*KRT2*	*Keratin2*	3	2304	1008	23.677	6.987	1	2304	1	0.017	0.005	1	−10.08	11.26
*KLK7*	*Kallikrein-related peptidase 7*	14	1547	220	7.696	2.271	1	1547	2	0.052	0.016	1	−6.98	9.93
*C2CD4C*	*C2 Calcium Dependent Domain Containing 4C*	5	9575	185	1.046	0.308	1	9575	4	0.017	0.005	1	−5.78	12.98
*SLC6A14*	*Solute Carrier Family 6 Member 14*	X	3254	140	2.328	0.687	1	3254	4	0.050	0.015	1	−5.63	12.26

**Table 5:** Upregulated DEGs with most extreme (positive) log_2_ fold change and −log_10_FDR values.

Gene ID	Gene Name		Ram O					Ram M					Log_2_ Fold Change	−Log_10_FDR
		Chr. Number	Length of Read (bp)	Reads	TPM	RPKM	Relative RPKM	Length of Read (bp)	Reads	TPM	RPKM	Relative RPKM		
*RPS12*	*Ribosomal protein S12*	8	512	2	0.212	0.062	1	511	717	5.793	17.637	1	8.11	13.43
*LOC114111581 (FTH1)*	*Ferritin heavy chain-like*	X	720	3	0.225	0.066	1	720	445	25.016	7.769	1	6.86	14.11

**Table 6:** Significant results from GO enrichment analysis for *KRT2* gene.

Gene ID	GO Term	Description	FDR
*KRT2*	0006996	Organelle organization	1.27 × 10^−9^
	0007010	Cytoskeleton organization	0.000022
	0045103	Intermediate filament-based process	0.002496
	0045104	Intermediate filament cytoskeleton organization	0.002496
	0045109	Intermediate filament organization	0.004209

## Data Availability

Data generated and analyzed during this study have been deposited in ZENODO (UE Open Research Repository) under doi:10.5281/zenodo.21355789.

## Abbreviations

The following abbreviations are used in this manuscript:

AI	artificial insemination
ANGRA	Asociación Nacional de Criadores de Ganado Ovino Selecto de Raza Rasa Aragonesa
AR	Acrosome-reacted sperm
C	Capacitated sperm
*C2CD4*	*C2 CalciumDependentDomainContaining 4C gene*
CENSYRA	Regional Centre for Animal Selection and Reproduction)
CFDA	Carboxyfluorescein diacetate
DEGs	differentially expressed genes
FDR	false discovery rate
H_2_DCFDA	2′,7′-dichlorodihydrofluorescein diacetate
IP	propidium iodide
IQR	interquartile range
*KLK7*	*Kallikrein-related pepetidase 7 gene*
KRT1	Keratin 1
KRT14	Keratin12
KRT17	Keratin17
*KRT2*	*Keratin 2 gene*
KRT5	Keratin 5
*LOC114111581 (FTH1)*	*Ferritin heavy chain like gene*
NC	Non-capacitated sperm
PS	phosphatidylserine
ROS	Reactive oxygen species
RPKM	Reads per Kilobase of exon model per Million mapped reads
*RPS12*	*Ribosomal protein S12 gene*
SD	standard deviation
*SLC6A14*	*Solute Carrier Family 6 Membre 14 gene*
TPM	Transcripts per Million
TUNEL	Terminal deoxynucleotidyl transferase dUTP Nick End Labeling
